# Post-intravitreal Injection Endophthalmitis Identified with Point-of-care Ultrasound

**DOI:** 10.5811/cpcem.2021.11.54515

**Published:** 2022-05-05

**Authors:** Vahe Zograbyan, Matthias Barden, Ami Kurzweil

**Affiliations:** * St. George’s University School of Medicine, Grenada, West Indies; † Eisenhower Health, Department of Emergency Medicine, Rancho Mirage, California

**Keywords:** endophthalmitis, ocular ultrasound, ultrasound, vitreous, ophthalmology

## Abstract

**Case Presentation:**

An 88-year-old female presented to the emergency department (ED) with complaints of painful vision loss four days after an intravitreal injection for her neovascular macular degeneration. Her right eye visual acuity was markedly diminished with an absence of red reflex. A point-of-care ocular ultrasound was performed and demonstrated hyperechoic vitreous debris concerning for endophthalmitis.

**Discussion:**

Endophthalmitis is an infection of the vitreous or aqueous humors commonly caused by exogenous sources, such as inoculation of bacteria into the eye from surgery, injections, or trauma. It is an ophthalmologic emergency as it is a vision-threatening infection. Although a rare complication, post-surgery or post-injection are the leading causes of endophthalmitis. Point-of-care ocular ultrasound findings suggestive of endophthalmitis, such as hyperechoic vitreous debris, aid in the timely diagnosis and treatment of patients in the ED.

## CASE PRESENTATION

An 88-year-old female with a past medical history of neovascular macular degeneration presented to the emergency department (ED) with complaints of right eye pain, worsening vision, floaters, and eye dryness since receiving an intravitreal, anti-vascular endothelial growth factor injection four days prior. On physical exam the patient’s right eye had crusted exudates on eyelid margins, conjunctival injection, and absent red reflex. Visual acuity was markedly diminished with 20/200 in the right eye and 20/100 in the left eye. Tetracaine ophthalmic drops were administered for topical analgesia to both eyes, and intraocular pressures were measured to be within normal limits. Fluorescein dye and Wood’s lamp exam found no uptake or corneal abrasion. A point-of-care ocular ultrasound demonstrated significant swirling echogenic debris within the posterior vitreous humor and attached, thickened retina (Image and Video). Normal retinal thickness is around 0.1–0.3 millimeters, although this is not commonly measured on point-of-care ocular ultrasound.^6^ All findings were concerning for endophthalmitis.

The patient’s ophthalmologist was consulted regarding the findings and recommended tobramycin-dexamethasone ophthalmic drops and cefazolin two grams intramuscular in the ED with a disposition for immediate follow-up at their office for intravitreal cultures and antibiotic administration.

## DISCUSSION

Endophthalmitis is a bacterial or fungal infection of aqueous or vitreous humors. Most cases of endophthalmitis are caused by exogenous sources: usually inoculation from surgery, injections, or trauma. Endogenous endophthalmitis is much less common and caused by seeding of the eye from the bloodstream, which then extends from the choroid to the vitreous humor. Most cases of acute endophthalmitis are caused by bacteria, and it is an ophthalmologic emergency as it is a vision-threatening infection.

Acute post-cataract surgery and post-injection are the most common causes of endophthalmitis.^1^ Neovascular macular degeneration, as with our patient, is commonly treated with monthly intravitreal injections of anti-vascular endothelial growth factor medications. Although post-injection endophthalmitis is rare (0.09% per injection),^2^ patients receiving these monthly injections are increasingly vulnerable due to cumulative risk. Ocular ultrasound plays an important role in timely diagnosis of this vision-threatening complication and is easily applied in the ED setting.^3^,^4^,^5^

The differential diagnosis for mobile echogenic debris within the posterior vitreous chamber is broad. Common pathologies seen are vitreous hemorrhage, vitreous detachment, retinal detachment, foreign bodies, and lens dislocations. Some rare findings can include inflammatory or infectious etiologies such as intermediate uveitis, vitritis, or endophthalmitis.^9^ The patient’s risk factors and clinical presentation lend to narrowing the diagnosis. Although there are no specific test characteristics for detecting endophthalmitis, ocular ultrasound has been shown to have sensitivities between 81.9–96.9% and specificities ranging from 82.3–96.3% for the diagnosis of vitreous hemorrhage and retinal detachments.^7^,^8^ These pathologies, especially vitreous hemorrhage, have similar appearances on ocular ultrasound. Treatment of endophthalmitis includes intravitreal cultures and empiric antibiotics with vitrectomy reserved for severe cases.

**CPC-EM Capsule**

What do we already know about this clinical entity?

*Endophthalmitis is a rare vision-threatening emergency that may occur after an intravitreal injection or ophthalmologic surgery*.

What is the major impact of the image(s)?

*The ocular ultrasound images display the common findings and characteristics in a patient with endophthalmitis. If not identified and treated in a timely manner, irreversible blindness may occur*.

How might this improve emergency medicine practice?

*Given that ultrasound is readily available, safe, and fast; recognition of ocular ultrasound findings may improve patient care through timely diagnosis and treatment of endophthalmitis*.

## Supplementary Information

Video Point-of-care ocular ultrasound demonstrating mobile echogenic debris within the vitreous chamber in a patient with endophthalmitis.

## Figures and Tables

**Image:**
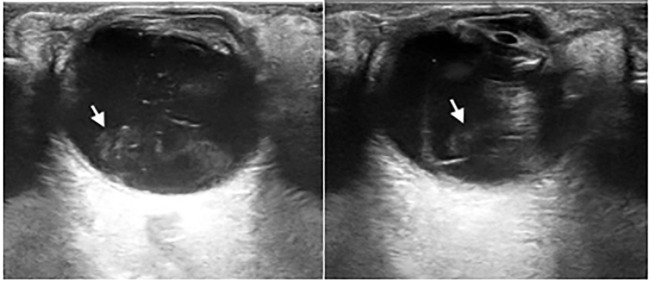
Point-of-care ocular ultrasound demonstrating hyperechoic debris (white arrows) within the vitreous chamber in a patient with endophthalmitis.

## References

[1] Durand ML, Miller JW, Young LH, Das T (2018). Endopthalmitis: An Overview. Endophthalmitis.

[2] Day S, Acquah K, Mruthyunjaya P (2011). Ocular complications after anti–vascular endothelial growth factor therapy in Medicare patients with age-related macular degeneration. Am J Ophthalmol.

[3] Kohanim S, Daniels AB, Huynh N (2012). Utility of ocular ultrasonography in diagnosing infectious endophthalmitis in patients with media opacities. Semin Ophthalmol.

[4] Kilker BA, Holst JM, Hoffmann B (2014). Bedside ocular ultrasound in the emergency department. Eur J Emerg Med.

[5] Tucker J, Patane J, Lahham S (2018). Point-of-care ultrasound detection of endophthalmitis. J Educ Teach Emerg Med.

[6] Chan A, Duker JS, Ko TH (2006). Normal macular thickness measurements in healthy eyes using Stratus optical coherence tomography. Arch Ophthalmol.

[7] Gottlieb M, Holladay D, Peksa GD (2019). Point-of-care ocular ultrasound for the diagnosis of retinal detachment: a systematic review and meta-analysis. Acad Emerg Med.

[8] Lahham S, Shniter I, Thompson M (2019). Point-of-care ultrasonography in the diagnosis of retinal detachment, vitreous hemorrhage, and vitreous detachment in the emergency department. JAMA Netw Open.

[9] Shah R, Rychwalski P, Kurzweil A (2021). Ocular point-of-care ultrasound: description of intermediate uveitis in an adolescent female. Pediatr Emerg Care.

